# Chemotherapeutic drugs stimulate the release and recycling of extracellular vesicles to assist cancer cells in developing an urgent chemoresistance

**DOI:** 10.1186/s12943-019-1114-z

**Published:** 2019-12-12

**Authors:** Xiaokun Wang, Dongjuan Qiao, Likun Chen, Meng Xu, Shupeng Chen, Liyan Huang, Fang Wang, Zhen Chen, Jiye Cai, Liwu Fu

**Affiliations:** 1 State Key Laboratory of Oncology in South China, Collaborative Innovation Center for Cancer Medicine, Sun Yat-sen University Cancer Center, Guangzhou, 510060 China; 2 Department of Chemistry, College of Life Science and Technology, Jinan University, Guangzhou, China

**Keywords:** Extracellular vesicles, Intercellular transfer, Multidrug resistance, ABCB1, Chemotherapeutic drugs

## Abstract

**Background:**

Chemotherapy is a widely used treatment for cancer. However, the development of acquired multidrug resistance (MDR) is a serious issue. Emerging evidence has shown that the extracellular vesicles (EVs) mediate MDR, but the underlying mechanism remains unclear, especially the effects of chemotherapeutic agents on this process.

**Methods:**

Extracellular vesicles isolation was performed by differential centrifugation. The recipient cells that acquired ATP-binding cassette sub-family B member 1 (ABCB1) proteins were sorted out from co-cultures according to a stringent multi-parameter gating strategy by fluorescence-activated cell sorting (FACS). The transfer rate of ABCB1 was measured by flow cytometry. The xenograft tumor models in mice were established to evaluate the transfer of ABCB1 in vivo. Gene expression was detected by real-time PCR and Western blotting.

**Results:**

Herein, we show that a transient exposure to chemotherapeutic agents can strikingly increase Rab8B-mediated release of extracellular vesicles (EVs) containing ABCB1 from drug-resistant cells, and accelerate these EVs to circulate back onto plasma membrane of sensitive tumor cells via the down-regulation of Rab5. Therefore, intercellular ABCB1 transfer is significantly enhanced; sensitive recipient cells acquire a rapid but unsustainable resistance to evade the cytotoxicity of chemotherapeutic agents. More fascinatingly, in the xenograft tumor models, chemotherapeutical drugs also locally or distantly increase the transfer of ABCB1 molecules. Furthermore, some Non-small-cell lung carcinoma (NSCLC) patients who are undergoing primary chemotherapy have a rapid increase of ABCB1 protein in their monocytes, and this is obviously associated with poor chemotherapeutic efficacy.

**Conclusions:**

Chemotherapeutic agents stimulate the secretion and recycling of ABCB1-enriched EVs through the dysregulation of Rab8B and Rab5, leading to a significant increase of ABCB1 intercellular transfer, thus assisting sensitive cancer cells to develop an urgent resistant phenotype. Our findings provide a new molecular mechanism of how chemotherapeutic drugs assist sensitive cancer cells in acquiring an urgent resistance.

## Introduction

Chemotherapy is one of the major choices against a variety of cancers. However, its effectiveness is often limited by the acquisition of multidrug resistance (MDR) in cancer cells [1]. For decades, the mechanistic researches primarily focus on investigating how cancer cells are endowed with intrinsic resistance or acquire a long-term durable resistance. Some key factors behind the response to chemotherapy have been characterized [2]. In these studies, MDR, whether pre-existing or acquired, is largely believed as a stable and heritable process. However, existing MDR theories cannot explain why some patients rapidly develop the resistance to therapy. There is also no reasonable explanation for the clinical phenomenon of resensitization to previous chemotherapy [3]. These phenomenons imply an unstable or transient and non-heritable resistant mechanism, which is rare to be known.

Cell to cell communication is extremely essential for orchestration and coordination of cellular events [4]. A new phenomenon of cellular communication through exchange of proteins or intact membrane patches has been demonstrated to commonly occur in biology [5, 6]. Emerging evidence shows that cell membrane proteins can transfer between cells both in vitro and in vivo [7]. These intercellular protein transfers can contribute to the pathological or physiological functions of recipient cells [8, 9]. To date, the importance of such a cellular cross-talk is being well understood, but the mechanisms and functional consequences of these transfers are still elusive. Extracellular vesicles (EVs), lipid bilayer-enclosed structures that contain a variety of proteins, mRNA and microRNAs, have been recognized as critical mediators of intercellular communication between cells in local and distant microenvironments. Many investigations have demonstrated an association between EV release and the development of MDR. The release of EVs can act as a protective mechanism for cell survival [10, 11]. Inhibition of EV release has the potential to be considered as an intervention strategy for circumvention of MDR. During the course of chemotherapy, tumors can acquire MDR to chemotherapeutic drugs through the induction of *ABCB1* gene expression [12–15]. Recent studies have proposed another potential mechanism by which cancer cells acquire MDR, which is intercellular transfer of ABCB1 [16–18]. Nevertheless, the significance and mechanism of ABCB1 intercellular transfer in clinical MDR is poorly understood.

From a clinical standpoint, it will be of utmost importance to elucidate the mechanism of how the cancer cells evade promptly chemotherapeutic treatment. In the present study, we investigated the effects and potential mechanism of chemotherapeutical agents on the release and recycling of extracellular vesicles. Under the exposure of low-dose chemotherapeutic agents, how the sensitive cancer cells acquire an urgent resistance against cytotoxicity is also showed. These investigations will lend further support to develop a valid therapeutic strategy to alleviate the MDR phenotype for successful cancer treatment.

## Materials and methods

### Cell lines

The human oral epidermoid carcinoma KB cells and vincristine-selected ABCB1-overexpressing KBv200 cells, the human colon carcinoma cells S1, and the human embryonic kidney 293 T cells were cultured in RPMI-1640 or DMEM supplemented with 100 U/mL penicillin, 100 U/mL streptomycin, and 10% fetal bovine serum at 37 °C in a humidified atmosphere of 5% CO2.

### GFP vector construction and lentiviral transduction

KB and S1 cells were transfected with lentivirus vectors carrying green fluorescent protein (GFP). The GFP sequence was cloned into the EcoR I and BamHI sites of the pSin4 vector, thus permitting continuous GFP expression. The 293 T cells were seeded into 10-cm cell culture dishes and cultured for 24 h prior to transfection. The recombinant lentiviral vector encoding GFP and the psPAX2 packaging plasmid and pMD2.G envelope plasmid were co-transfected into 293 T cells with lipofectamine TM 2000 reagent according to the manufacturer’s instructions. After 6 h transfection, the cell culture medium was replaced with fresh complete medium. After 48 h transfection, the culture medium was collected and centrifuged at 4000×g at 4 °C for 10 min to remove any cellular debris. The supernatant was filtered through a 0.45-μm filter into culture medium of KB or S1 cells which were seeded prior to infection. Transfected cells were selected with 1 μg/ml puromycin for one week. The stable cell lines were continuously cultured in culture medium supplemented with 0.2 μg/ml puromycin.

### Patient samples

Before and after primary chemotherapy, the peripheral blood was obtained from 21 patients with new diagnosed non-small cell lung cancer (NSCLC) after informed consent and with the approval of the ethics review Committee at Sun Yat-Sen University (Guangzhou, China). The monocytes were isolated using Ficoll–Hypaque density gradient centrifugation and cultured in RPMI1640 medium containing 20% FBS for further assay.

### Quantitative determination of transfer rate of ABCB1 by flow cytometry

The phycoerythrin (PE)-conjugated monoclonal antibody (mAb) against ABCB1 (PE-ABCB1) and isotype control antibody (PE-labeled mouse IgG2a) were used in the quantitative flow cytometric assays. Before detection, the pretreated or untreated KB-GFP cells were co-cultured with KBv200 cells or EVs for 72 h, with or without the exposure to various agents. Then single-cell suspensions were prepared by the addition of 0.5 mM EDTA followed by three washes with an isotonic PBS buffer [supplemented with 0.5% bovine serum albumin (BSA)]. Cells were then co-incubated at 4 °C for 45 min with PE-conjugated mAb against ABCB1. After washing, cells were resuspended in 400 μL PBS buffer for flow cytometric analysis. The relative transfer rate (TR) of ABCB1 was calculated by the following formula:

Transfer rate (%) = (sensitive cells that acquired ABCB1/total sensitive cells) × 100%.

### Establishment of in vivo xenograft tumor models

In vivo experiments were done in accordance with the guidelines for the use of laboratory animals of the Sun Yat-Sen University Institutional Animal Care and Use Committee. The mice model for local transfer was established by subcutaneously inoculating a mixture of ABCB1 negative KB cells and ABCB1 positive KBv200 cells at a ratio of 1:1 into the flank of athymic nude mice (BALB/c-nu/nu, 5-to 6- week-old, *n* = 10). The systemic transfer model was generated by subcutaneously injecting GFP positive KB cells and KBv200 cells into the contralateral armpit of forelimb, respectively (*n* = 10). The unilateral KB and KBv200 cell xenograft tumor models were also established as controls (*n* = 5). The mice of each xenograft models were randomized into two groups (5 in each group) after the tumors reached a mean volume of about 100 mm^3^, and then received treatments: (i) saline (every 3 days for 5 times, i.p.); (ii) cisplatin (every 3 days for 5 times, 2 mg/kg, i.p.). At the end of the experiment, the mice were euthanized and the tumors were excised for further analysis.

### Isolation and detection of extracellular vesicles

Extracellular vesicles isolation was conducted from the culture media of 75% confluent KBv200 cells by differential centrifugation. Briefly, cell supernatants were isolated and centrifuged at 2000 g for 5 min to remove floating cells and debris. Cell-free supernatant was then transferred in ultracentrifuge tubes and spun down at 20,000 g for 30 min. The supernatants were then further ultra-centrifugated at 100,000 g for 1 h. After the removal of supernatants, the pellet was resuspended in PBS which was further centrifuged at 100,000 g for 1 h. The final pellet was either resuspended in RPMI-1640 to co-culture with KB cells or lysed for protein extraction. The EVs that were used for co-culture was quantified to protein concentrations with a standard BCA protein assay methods. For fluorescence labeling, DiI solution (1 μM as a working concentration, Beyotime) or FITC-labeled annexin V was added directly to EV suspensions. After incubation for 20 min (DiI) or 45 min (annexin V), the numbers of EVs were analyzed with Beckman Coulter’s MoFlo XDP cell sorter. The same time period 1 min was used for reference. The DiI dye was excited at 549 nm.

For the sucrose density gradient centrifugation, isolated EV pellets were resupended in PBS and layered on 10–80% sucrose density gradient stock solutions which were formed in polypropylene centrifugation tubes (Beckman Coulter, USA). After centrifugation at 100, 000 g, 4 °C for 24 h, the fractions were aliquoted and analyzed by Western blotting.

### Transmission electron microscopy (TEM)

Cell culture media were collected from KBv cells with or without VCR treatment. The EVs pellets were isolated as described above and resuspended in 2% paraformaldehyde. Then the EVs suspensions were mounted on copper grids, fixed by 1% glutaraldehyde in cold PBS for 5 min, washed in sterile distilled water 2 min for 8 times. Afterwards, the samples were contrasted by ranyl-oxalate solution at pH 7.0 for 5 min and embedded by methyl cellulose-UA solution for 10 min on ice. After removal of excess cellulose, the samples were air-dried for 5–10 min. A JEM-1400 transmission electron microscope was used to image EVs samples at a voltage of 120 kV.

### Immunofluorescence analyses

Cells were seeded on 13 mm glass coverslips placed in the wells of a 24-well plate. After co-incubation, cells were washed with phosphate-buffered saline (PBS) and fixed with 4% paraformaldehyde for 5 min at room temperature followed by permeabilized with 0.1% Triton X-100. After washing three times with PBS, cells were blocked with 1% BSA for 30 min and incubated overnight at 4 °C with mAbs against ABCB1 diluent in 1% BSA. Then Cy3-labled goat anti-mouse IgG (11000) was added and incubated at room temperature in dark for 1 h. Finally, the slides were washed and imaged using a FV1000 confocal microscope (Olympus, Tokyo, Japan).

For the immunofluorescence of xenograft tumors, the paraffin-embedded xenograft tumor blocks were first sectioned in 4 μm slices and placed on slides. Then the sections were heated at 60 °C for 20 min following deparaffination with 100% xylene (5 min, twice). After hydration with graded ethanol (100% ethanol, 95% ethanol, 80% ethanol, and 70% ethanol), the slides were intermittently heated in 0.1 M citrate buffer using microwave oven for 30 min to retrieve the antigens. After washing with PBS-Tween 20, the slides were blocked in 5% BSA for 30 min followed by incubation with primary antibody against ABCB1 (1:200) overnight at 4 °C. The slides were rinsed in PBS-Tween 20, and incubated with Cy3-conjugated goat anti-mouse IgG (1:1000) at room temperature in dark for 1 h. DAPI was used to stain the cell nuclei (blue) by incubating the slides at room temperature for 5 min. Finally, the glass coverslips were mounted onto the slides for analysis. The images were photographed using a FV1000 confocal microscope (Olympus, Tokyo, Japan).

### Doxorubicin (DOX) and Rhodamine 123 (Rho123) accumulation assay

The logarithmically growing KB or KBv200 cells (2 × 10^6^/ml) were incubated with 5 μg/ml Rho-123 for 1 h or 10 μM Dox for 3 h at 37 °C in RPMI-1640 medium, respectively. After incubation, cells were collected and washed twice with ice-cold PBS. Finally, the cells were resuspended in PBS buffer for flow cytometric analysis (Beckman Coulter, Cytomics FC500, USA). The relative values were identified by dividing the fluorescence intensity of each measurement by that of control cells.

### Enzyme-linked immunosorbent assay (ELISA)

KBv200 cells were treated with various concentrations of vincristine (VCR) for 72 h, the supernants were collected and centrifuged for 15 min at 2000 g/min to remove the dead cells and debris. The expression levels of ABCB1 protein were measured by ELISA kit (Cloud-Clone Corp) according to manufacturer’s instructions.

### Cell viability assay

Cells were harvested during logarithmic growth phase and seeded in 96-well plates at a density of 1.0 × 10^4^ cells/mL in a final volume of 190 μL/well. After 24 h incubation, 10 μL of Dox or DDP at a full range of concentrations was added to 96 -well plates. After 68 h incubation, 10 μL MTT solutions were added directly to each well. The plates were incubated for additional 4 h at 37 °C incubator. Subsequently, the supernatant was removed, and MTT crystals were solubilized with 100 μL anhydrous DMSO each well. Thereafter, cell viability was measured by model 550 microplate reader (Bio-Rad) at 540 nm, with 655 nm as reference filter. The 50% inhibitory concentration (IC50) was determined as the anticancer drug concentration causing 50% reduction in cell viability and calculated from the cytotoxicity curves using Bliss method. Experiments were performed at least three times.

### Quantitative reverse transcription-PCR

Total cellular RNA was isolated from different experimental group cells using the Trizol reagent (Invitrogen, USA), and subjected to reverse transcription-PCR. Real-time PCR was performed with a Bio-Rad CFX96 real-time system (BIO-RAD, Hercules, CA, USA). The amount of each target gene in a given sample was normalized to the level of GAPDH in that sample. The 2-ΔΔCT method was used to analyze the relative changes in gene expression. The PCR primers used in this study were summarized in Additional file 4: Table S1.

### RNA interference

The siRNAs specific for human caveolin-1 and Rab5 were customarily synthesized by Shanghai Gene-Pharma Co., Ltd. (China). Lentiviral shRNA vectors targeting clathrin and dynamin 2 were purchased from Shanghai GeneChem Co., Ltd. (China). Lentiviral shRNA vectors targeting Rab8B were constructed according to standard protocol with minor modifications. The siRNAs or shRNAs were transfected into the KB cells at 70–80% confluent using Lipofectamine 2000 (Invitrogen) according to the manufacturer’s instructions. The expression levels of targeted proteins were determined after 48 h by Western blotting analysis. The efficient targeting sequences for specific genes were shown in Additional file 4: Table S2. The scrambled sequence (s.c.) was used as control.

### Statistics

The statistical significance of differences was assessed using a 2-tailed Student’s t test or nonparametric statistics (SPSS16.0) for comparison between groups. Values represent mean ± SD. For all tests, the significance was determined at *p* < 0.01 (**) and *p* < 0.05 (*).

## Results

### Chemotherapeutic drug promotes the release of extracellular vesicles in resistant cells

**Fig. 1:**
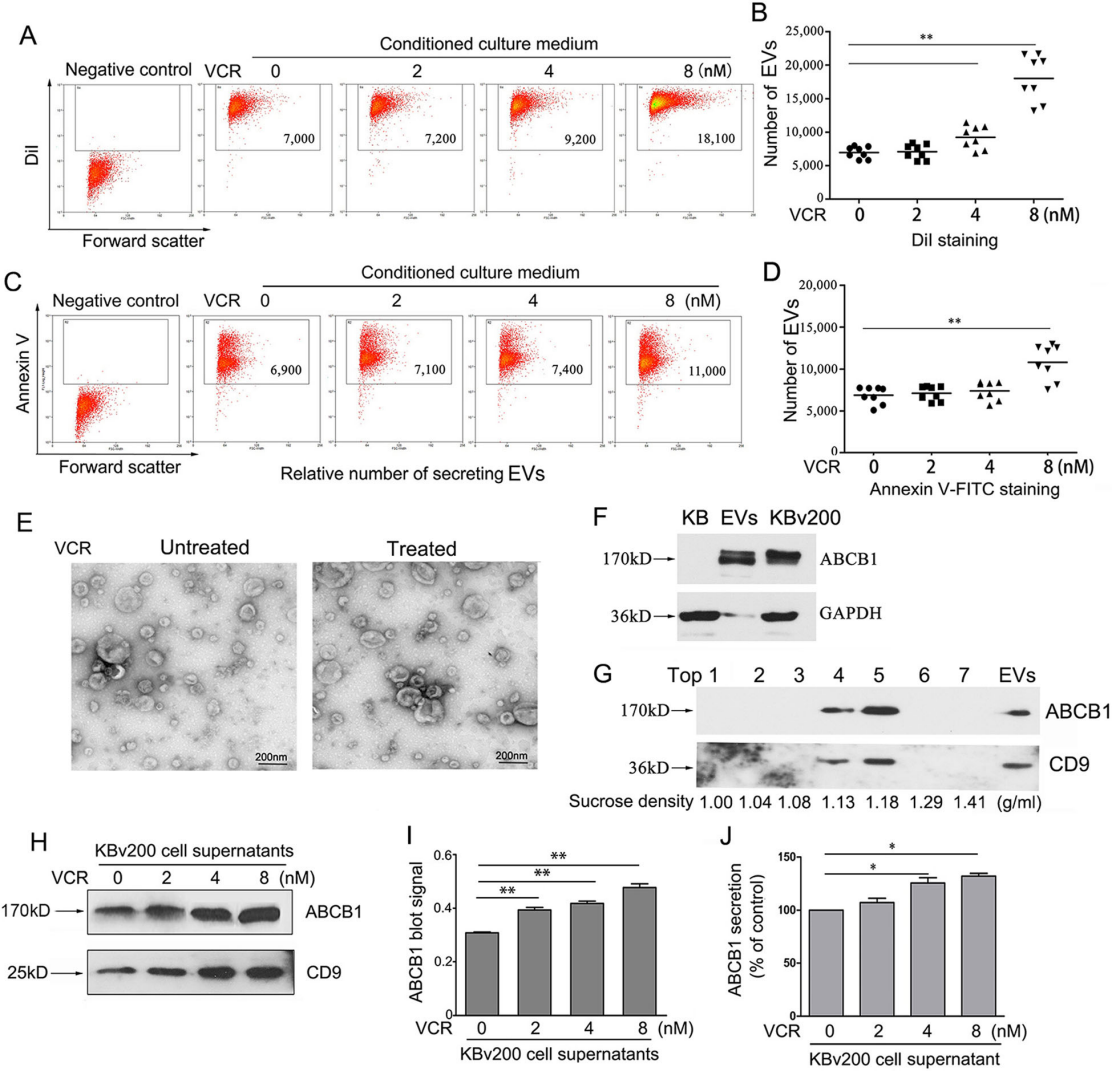
Chemotherapeutic drug VCR stimulates the secretion of ABCB1-enriched EVs. **A-B** The stimulation of chemotherapeutic agent VCR on the EV release from the resistant KBv200 cells was showed. **A** Representive dot plots by flow cytometry. **B** Quantification of EVs. Horizontal lines represent the means from eight detections. **C-D** VCR promoted the EVs secretion in resistant KBv200 cells detected by flow cytometry with labeling FITC-conjugated Annexin V. **E** TEM images for EVs from VCR-untreated and treated KBv200 cells. **F** ABCB1 proteins were detected in EVs. **G** Sucrose density gradient centrifugation of EVs. **H-I** VCR obviously increases the extracellular ABCB1 protein level of KBv200 cells determined by Western blotting. **J **Enzyme-linked immunoabsorbent assay (ELISA) shows VCR dose-dependently enhances the ABCB1 secretion in KBv200 cells

Previous study showed that chemoresistant cancer cells express more membrane shedding-related genes compared with chemosensitive cell [10, 19]. We first investigated the effect of low-dose chemotherapeutic agent vincristine (VCR) on the release of EVs in resistant KBv200 cells. Our previous study showed that IC50 of VCR in KBv200 cells was 1.318 ± 0.256 μM [20]. We chose 2, 4, and 8 nM as a working concentration at which 90% cells survived. The equal amounts of cell culture supernatants from same number of KBv200 cells that were exposed to various concentrations of VCR were collected. The secreted EVs were purified from cell supernatant by ultracentrifugation, followed by detecting the amount of EVs by a relative semi-quantitative flow cytometry assay after labeling with a membrane probe DiI dye. As shown in Fig. 1, a-b, low concentrations of VCR can significantly stimulate resistant KBv200 cells to secrete more EVs. Likewise, we used flow cytometry to study the release of annexin V-binding membrane EVs [21]. A dramatic increase in annexin V-binding EV counts was also observed in the media of KBv200 cells after exposure to 8 nM VCR (Fig. 1, c-d). We isolated EVs with a standard differential ultracentrifugation-based method from VCR- treated and untreated KBv200 cells, respectively, and characterized these EVs using transmission electron microscope (TEM). The TEM images showed that VCR did not seem to affect the shapes of EVs (Fig. 1, e). The EVs presented a cup-shaped or round-shaped, membrane-enclosed structures. And these purified EVs were rich in ABCB1 proteins detected by Western blotting (Fig. 1, f). To further clarify whether ABCB1 is contained in EVs, we performed sucrose density gradient ultracentrifugation followed by Western blotting. The results showed that both ABCB1 and CD9 were present in EVs which migrated to a specific sucrose density (1.13–1.18 g/ml) (Fig. 1, g). These data demonstrated the presence of ABCB1 in EVs. It was noteworthy that VCR obviously increased the level of extracellular ABCB1 protein by Western blotting (Fig. 1, h-i). The elevated CD9 protein (a marker of EVs) also indicated an increase in the amount of secreted EVs (Fig. 1, h). In addition, an enzyme-linked immunoabsorbent assay (ELISA) confirmed a dose-dependent increment in ABCB1 secretion induced by VCR (Fig. 1, j). Collectively, these observations clearly suggest that low concentrations of chemotherapeutic drug VCR stimulate resistant KBv200 cells to release more EVs containing ABCB1.

### EVs can be functionally internalized by sensitive cancer cells in a dose-dependent manner

**Fig. 2:**
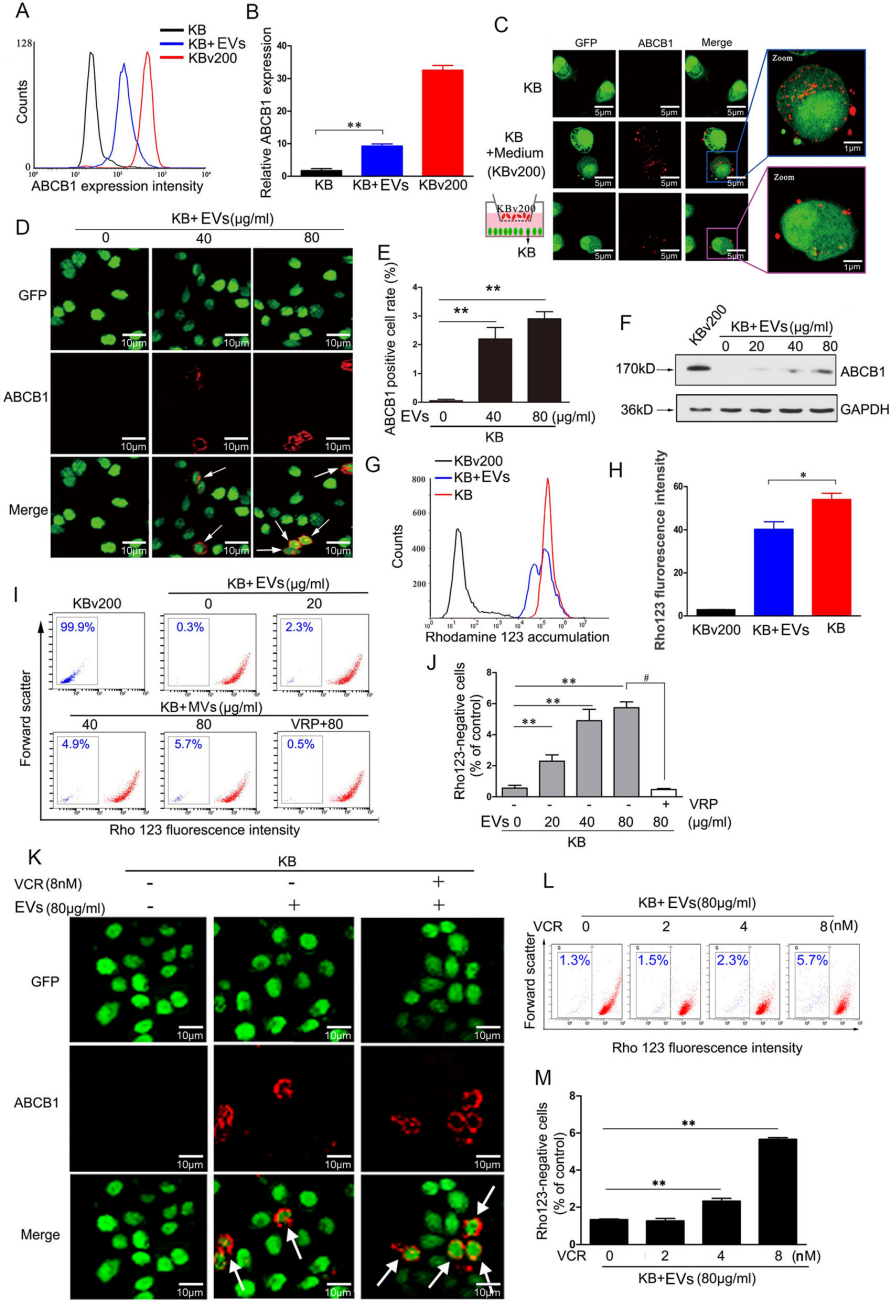
Membrane extracellular vesicles are dose-dependently transferred to sensitive cancer cells. **A-B** KB cells acquire moderate ABCB1 surface expression after co-incubating with EVs. **C** Representative confocol images show the involvement of contact-independent transfer in the intercellular transfer of ABCB1. KB cells are co-cultured with upper culture media isolated from resistant KBv200 cells (middle panel), or co-cultured with KBv cells but separated by a permeable membrane (1.0-μm pore size) to allow the traffic of secreted proteins but exclude direct cellular contact (lower panel). Red represents Cy3-ABCB1 and green represents GFP. **D-E** Representative confocol images show the increment of anchoring amount of ABCB1 to KB cells with the increasing EVs amount in co-cultured system. **F** The expression level of ABCB1 in KB cells is elevated with the increasing amount of EVs in co-cultures. **G-H** The accumulation of Rho123 in KB cells was reduced after incubation with EVs. **I-J** The co-incubation of KB cells with EVs from KBv200 cells dose-dependently endows sensitive KB cells with the ability to transport rhodamine 123. **K** The short-term exposure to 0.008 μM VCR results in a dominant plasma membrane localization of ABCB1 in recipient KB cells after cocultivation with equal amounts of EVs (80 μg/ml), as compared to untreated controls. **L-M** Flow cytometric analysis shows VCR resulted in an obvious concentration-dependent increase in the percentage of negative rhodamine 123 staining when KB cells are cocultured with equal EVs. Values are means ± SD. * = *P* < 0.05, ** = *P* < 0.01

In order to determine the effects of EVs on the development of drug resistance, we first confirmed the transfer of EVs carrying ABCB1 between cells. After co-incubating with collected EVs, drug-sensitive KB cells acquired moderate ABCB1 surface expression (Fig. 2, a-b). This suggests that EVs can be internalized by sensitive cancer cells. To verify this, we established stably expressing GFP drug –sensitive KB cells to perform a co-culture assay. ABCB1 was detectable in KB cells following cocultivation with upper culture media isolated from resistant KBv200 cells, but not in KB cells cultured alone (Fig. 2, c, top and middle panel). Furthermore, an in vitro transwell cell culture assay, in which KB and the resistant counterpart KBv200 cells were co-cultured but separated by a permeable membrane (1.0-μm pore size) to allow the traffic of secreted proteins but exclude direct cellular contact, also showed that KB cells acquired ABCB1 expression (Fig. 2, c, bottom panel). More importantly, the acquisition of ABCB1 proteins in KB cells was susceptible to the amounts of EVs. Because the number of ABCB1 positive KB cells was elevated with the increasing EVs amount in co-cultured system (Fig. 2, d-e). In addition, Western blotting showed a dose-dependent increase of ABCB1proteins in KB cells (Fig. 2, f). However, there was no detectable mRNA in KB cells even though incubation with 80 μg EVs (Additional file 1: Figure. S1, A). Taken together, it is concluded that sensitive KB cells could dose-dependently gain non-genetic ABCB1 protein expression by way of intercellular EVs transfer.

To verify whether the EVs internalized by KB cells are functional, we performed a Rhodamine 123 (Rho 123, a substrate of ABCB1) efflux assay. After KB cells were incubated with EVs, cellular Rho 123 fluorescence intensity was decreased compared to KB cells. This suggests that some KB cells obtain the ability to efflux Rhodamine 123 (Fig. 2, g-h). It is worthy of note that the ratio of KB cells with low Rhodamine 123 fluorescence was increased in a dose dependent manner after co-incubation with EVs (Fig. 2, i-j). However, in the presence of 10 μM verapamil (VRP, an inhibitor of ABCB1), the ratio of low Rho 123 fluorescent KB cells was significantly reduced (Fig. 2, i-j). These data showed ABCB1 proteins which were acquired by the internalization of EVs in KB cells could serve the function of efflux pump.

### Chemotherapeutic agents facilitate the recycling of EVs

Due to ABCB1 is a 170 kDa transmembrane glycoprotein, partial KB cells acquire the ability of pumping Rho123 out from the cells also indicated that these internalized EVs were recycled. We then investigated the effect of chemotherapeutic agents on the recycling of EVs in recipient cells. As showed in Fig. 2, k, the short-term exposure to 8 nM VCR resulted in a dominant plasma membrane localization of ABCB1 in KB cells after cocultivation with equal amounts of EVs (80 μg/ml), as compared to untreated controls. Consistent with this, treatment with VCR resulted in an obvious concentration-dependent increase in the percentage of KB cells with negative Rho 123 staining when the KB cells were cocultured with equal amount of EVs (Fig. 2, l-m). A similar result was obtained by the treatment with another chemotherapeutic drug DDP (Additional file 1: Figure. S1, B-C). These observations implied that chemotherapeutic drug induced an enhanced recycling back to plasma membrane of ABCB1 after EVs were taken up by recipient cancer cells.

### Chemotherapeutical agents strikingly promote the intercellular transfer of ABCB1

**Fig. 3:**
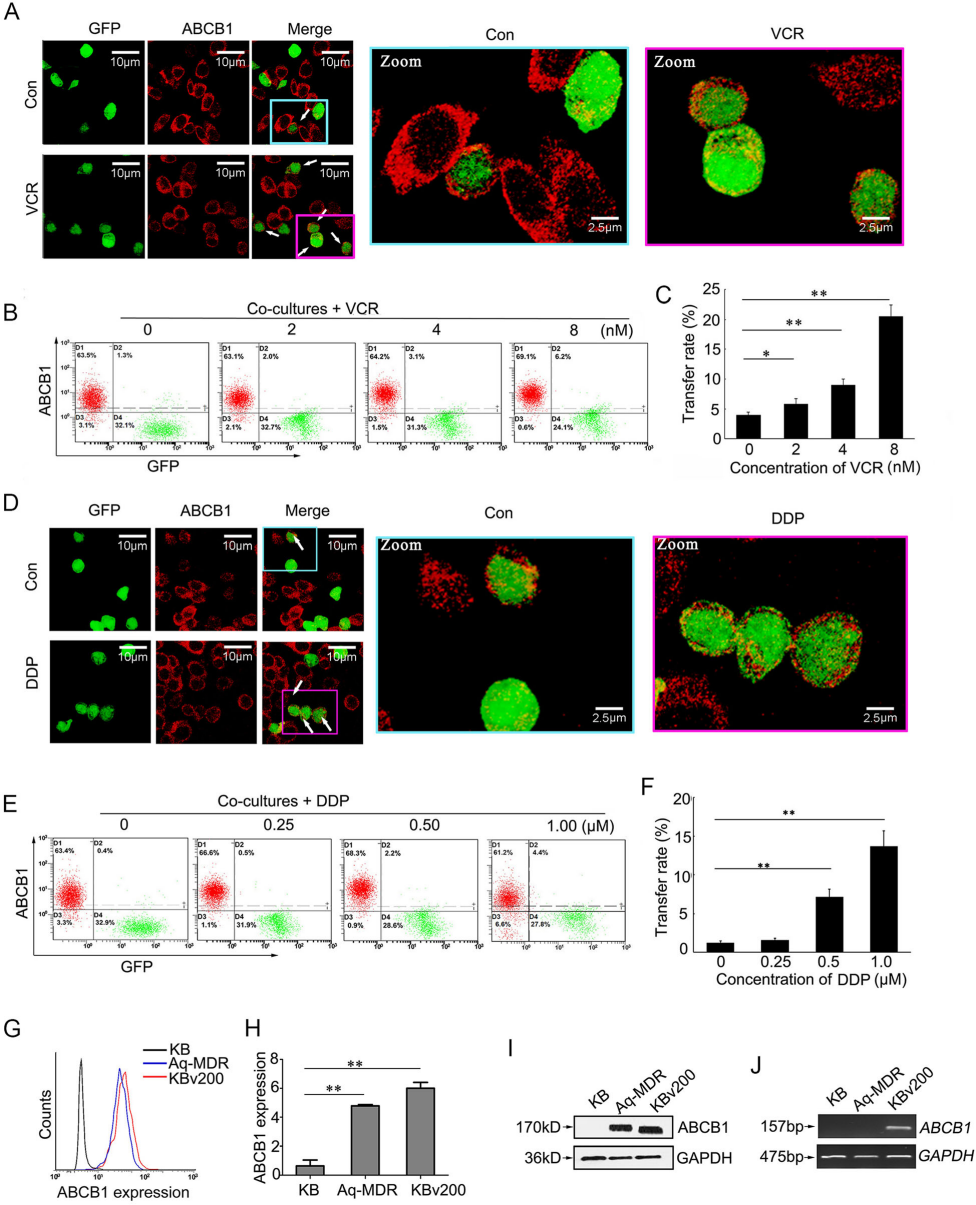
Chemotherapeutic drugs strikingly facilitate the intercellular transfer of ABCB1. **A** The representative confocal images show the promotion of VCR on the intercellular transfer of ABCB1. Red represents Cy3-ABCB1 and green is GFP. **B-C** The effect of VCR on the intercellular transfer of ABCB1 is determined by flow cytometry. **D** The representative confocal images show the DDP-increased intercellular transfer of ABCB1. **E-F** The effect of DDP on the intercellular transfer of ABCB1 is determined by flow cytometry. **G-H** Aq-MDR cells acquire moderate surface expression levels of ABCB1. **I-J** Aq-MDR cells express high level of ABCB1 protein, but not mRNA. GAPDH is used as an internal control. Data represent the mean transfer rate ± S.D. * = *P* < 0.05, ** = *P* < 0.01

Based on above findings, it is speculated that low concentrations of chemotherapeutical agents may promote the intercellular transfer of ABCB1 by stimulating the release and recycling of EVs. This was confirmed by a direct immunofluorescence analysis after co-incubation of GFP^+^ KB cells with KBv200 cells at a ratio of 2:1 with a short-term treatment with chemotherapeutic agents. It was observed that intercellular transfer of ABCB1 was significantly enhanced by VCR (Fig. 3, a). The transfer ratio of ABCB1 can be improved up to 20.5% when the co-cultures were exposed to 8 nM VCR (Fig. 3, b-c). To further confirm whether the stimulation of ABCB1 transfer by chemotherapeutic agents was specific or selective, we detected the effect of another two conventional chemotherapeutic drugs cisplatin (DDP) and doxorubicin (Dox), which has different chemical structures and therapeutic mechanisms. Surprisingly, both DDP and Dox were found to facilitate the intercellular transfer of ABCB1 (Fig. 3, d-f; Additional file 2: Figure. S2, A-B). These findings strongly showed that intercellular transfer of ABCB1 protein can be significantly promoted by conventional chemotherapeutic drugs regardless of whether the drugs are substrates of ABCB1.

To eliminate the possibility that, in the presence of chemotherapeutic drugs, the elevated ABCB1 expression levels in KB cells were, in fact, attributed to the induced expression of *ABCB1* gene, we performed the same experiments in pure sensitive KB cells. It was indicated that any of the used chemotherapeutic drugs, even at the highest concentrations, could not increase the surface expression of ABCB1 in sensitive KB cells, suggesting that chemotherapeutic drugs don’t induce the expression of ABCB1 gene in short-term culture (Additional file 2: Figure. S2, C-D). In addition, we used a digital cell sorter and a stringent multi-parameter gating strategy to physically sort out two subpopulations of GFP + PE- (sensitive KB cells) and GFP + PE+ (Aq-MDR cells) cells from co-cultures after staining with PE-UIC2 ABCB1 mAb. A surface expression analysis of ABCB1 by flow cytometry showed that Aq-MDR cells acquired moderate membrane surface expression of ABCB1 (Fig. 3, g-h). Western blotting verified that Aq-MDR cells gained a high expression of ABCB1 protein (Fig. 3, i). Nevertheless, there is no detectable ABCB1 mRNA in Aq-MDR cells (Fig. 3, j). In addition, an existence of ABCB1 intercellular transfer between GFP-expressing drug-sensitive human colon carcinoma cells S1 and human oral epidermoid carcinoma KBv200 cells was observed (Additional file 2: Figure. S2, E). These results confirmed a nongenetic mechanism by which intercellular transfer of ABCB1 from KBv200 cells to sensitive recipient cells.

### Extracellular vesicles-mediated ABCB1 intercellular transfer confers a transient resistant phenotype to sensitive cancer cells

**Fig. 4:**
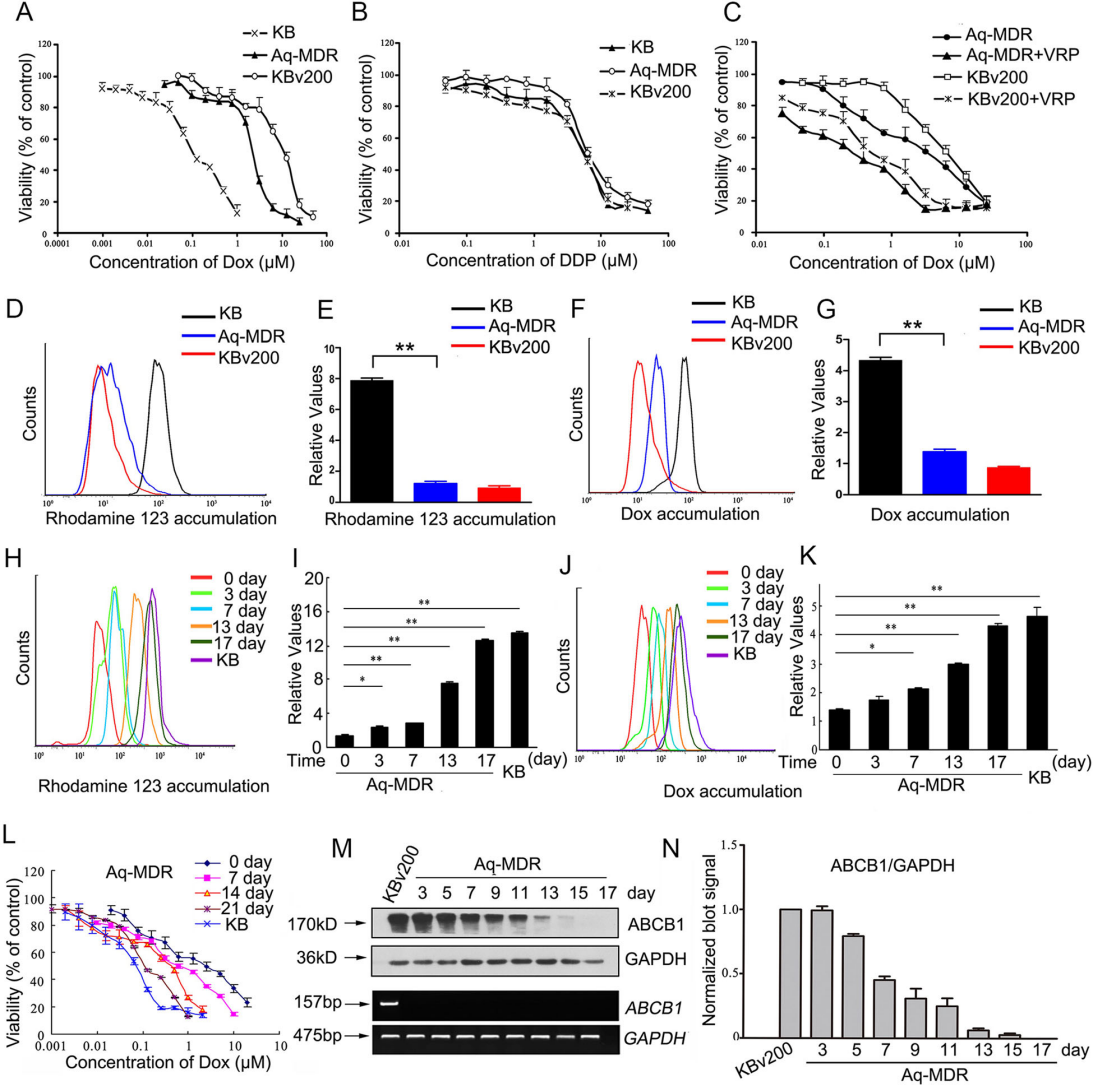
Extracellular vesicles-mediated ABCB1 protein transfer confers a transient resistant phenotype to sensitive cancer cells. **A-B** Cytotoxicity of Dox or DDP in the indicated cell lines is determined by MTT assay. **C** Effect of ABCB1 inhibitor verapamil on the cytotoxicity of Dox in the indicated cell lines is showed. **D-G** Intracellular accumulation of Rho-123 or Dox in the indicated cell lines is measured by flow cytometric analysis. **H-K** A long-term investigation of the accumulating level of Rho-123 or Dox in Aq-MDR cells is showed by flow cytometry. **L** The chemoresistance of Aq-MDR cells to Dox is declined gradually over time, monitoring by a long-term following MTT assay. **M-N** The protein expression of ABCB1 in Aq-MDR cells decreased with the cultured time, but mRNA level of ABCB1 did not alter. Values are means ± SD for three different experiments. * = *P* < 0.05, ** = *P* < 0.01

MTT assay showed that IC_50_ value of Dox in Aq-MDR cells was significantly increased compared with that in pure sensitive KB cells, indicating transferred ABCB1 was functional (Fig. 4, a; Additional file 3: Figure. S3, A). Whereas there was no significant difference in the IC_50_ values of DDP, which is not a substrate drug of ABCB1, between Aq-MDR cells and pure sensitive KB cells (Fig. 4, b; Additional file 3: Figure. S3, B). More importantly, the resistant phenotype mediated by transferred ABCB1 could also be reversed by verapamil, a well-known ABCB1 inhibitor (Fig. 4, c; Additional file 3: Figure. S3, C). Intracellular accumulation assay of Rhodamine 123 and Dox, both of which were fluorescent substrates of ABCB1, were also performed in sorted Aq-MDR. The results showed that the relative fluorescence intensity of Rho123 and Dox in Aq-MDR cells was decreased by 3.27 and 2.51-fold, respectively, compared with that in pure sensitive KB cells, indicating that transferred ABCB1 reduced both Rho123 and Dox retention in Aq-MDR cells (Fig. 4,d-g). These results suggested that transferred ABCB1 can protect KB cells from the cytotoxicity by pumping chemotherapeutic drugs that are substrates of ABCB1 out from cells.

More importantly, a follow-up analysis showed the intracellular accumulation levels of Rho-123 and Dox were both increased gradually with the extension of culture time, implying a time-dependent decrease in efflux activity of transferred ABCB1 (Fig. 4, h-k). The chemoresistance in Aq-MDR cells was also monitored by a long-term following MTT assay. It was found that the chemoresistance of Aq-MDR cells to Dox was also declined progressively over time (Fig. 4, l. Additional file 3: Fig. S3, D). In addition, a long-term tracking Western blotting assay verified that the protein expression of ABCB1 in Aq-MDR cells was progressively reduced with the prolonging of time, probably because of either redistribution within daughter cells or proteolysis in the process of long-term culture (Fig. 4, m-n). However, no significant increase or decrease in ABCB1 mRNA was detected in Aq-MDR cells, when cultured in the absence of cytotoxic drugs; even though for a long time (Fig. 4, m). Therefore, ABCB1 protein molecules that transferred to Aq-MDR cells were not permanently stable, and more probably was a temporary or homeostatic process.

### Dysregulation of Rab8B and Rab5 play a key role in the chemotherapeutical agents-promoted intercellular transfer of ABCB1

**Fig. 5:**
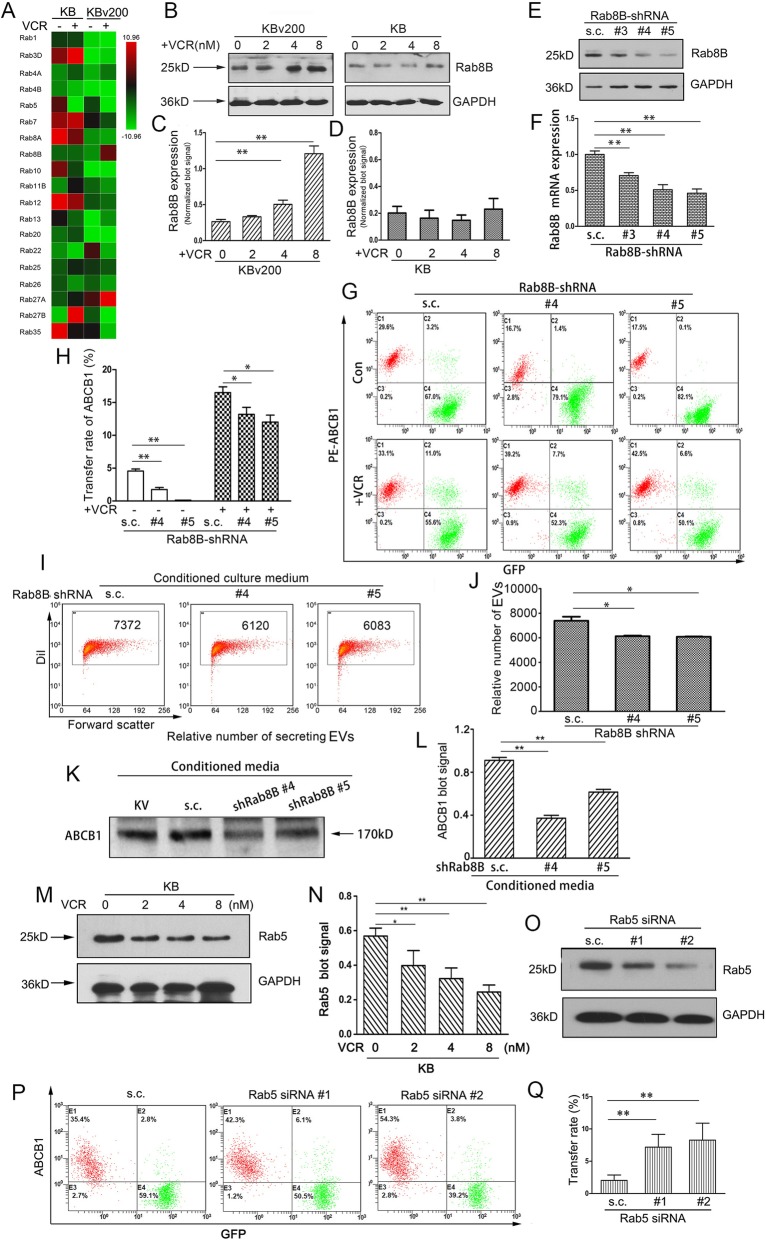
Upregulation of Rab8B induced by VCR resulted in the increase of ABCB1-enriched EVs secretion. **A** Human Rab GTPase family members targeting trafficking of vesicles were screened using real-time quantitative PCR. The Rabs that were significantly upregulated or downregulated (*P* < 0.01, > 5 fold change) by the treatment of VCR in KBv200 cells were identified and consider for further validation. **B-D** The treatment with VCR led to a significant increase in Rab8B protein expression level in KBv200 cells. **E-F** The silence of Rab8B by three different shRNAs was validated by Western blotting and qPCR, respectively. Scramble shRNA sequence was used as a control (termed as s.c.). **G-H** Inhibition of Rab8B reduced the rate of ABCB1 intercellular transfer. ** I-J** Inhibition of Rab8B resulted in a reduction in the secretion of MVs. **K-L** Silencing of Rab8B dramatically reduced the secretion of ABCB1 proteins. **M-N ** Immunoblot shows VCR suppresses the expression of Rab5 in KB cells. ** O** Endogenous Rab5 in KB cells is knockdown by specific siRNA against Rab5 as compared to control scramble sequence. **P-Q** The transfer rate of ABCB1 is increased when the expression of Rab5 is suppressed in KB cells. * = *P* < 0.05, ** = *P* < 0.01

In order to identify the factors that regulate the release of EVs promoted by chemotherapeutic agent VCR, we performed a screening aiming at Rab GTPase family which is critical for extracellular vesicles formation and secretion using quantitative real-time PCR. Rab8B was identified as a strong regulator in donor KBv200 cells under the exposure of VCR (Fig. 5, a). The results of Western blotting validated that the treatment with VCR led to a significant increase in Rab8B protein expression level in KBv200 cells, but not in KB cells (Fig. 5, b-d). To understand the contribution of Rab8B towards the release of ABCB1-containing EVs, we silenced the expression of Rab8B in KBv200 cells by three different shRNAs, and evidenced the reduction of Rab8B by Western blotting and qPCR, respectively (Fig. 5, e-f). Two shRNAs targeting Rab8B that led to the greatest downregulation were used for further assay. We found the intercellular transfer of ABCB1 was significantly inhibited after Rab8B expression was reduced, either in the presence or absence of chemotherapeutic agents (Fig. 5, g-h). We measured the EVs secretion using the methodology described above and found the inhibition of Rab8B resulted in a slight reduction in the secretory amount of EVs (Fig. 5, i-j). In consistent with this, silencing of Rab8B dramatically reduced the secretion of ABCB1 proteins (Fig. 5, k-l). These data showed that up-regulation of Rab8B in KBv200 cells upon the treatment of VCR was responsible for the enhanced intercellular transfer of ABCB1 by regulating the release of EVs.

In recipient KB cells, we found that the mRNA level of Rab5 was decreased (Fig. 5, a). We pretreated KB cells with various concentrations of VCR for 72 h, and then performed Western blotting analysis. The results showed that VCR obviously inhibited the protein expression of Rab5, which mainly located in early endosomes (Fig. 5, m-n). When Rab5 was efficiently knocked down by two siRNAs in KB cells, prior to cocultivation with KBv200 cells, the intercellular transfer rate of ABCB1 was significantly elevated (Fig. 5, o-q). These findings suggest that downregulation of Rab5 is involved in chemotherapeutic drug-promoted recycling of EVs, thus affecting the intercellular transfer of ABCB1.

### Dynamin 2 and Clathrin mediate the endocytosis of ABCB1-containing EVs in recipient cancer cells

**Fig. 6:**
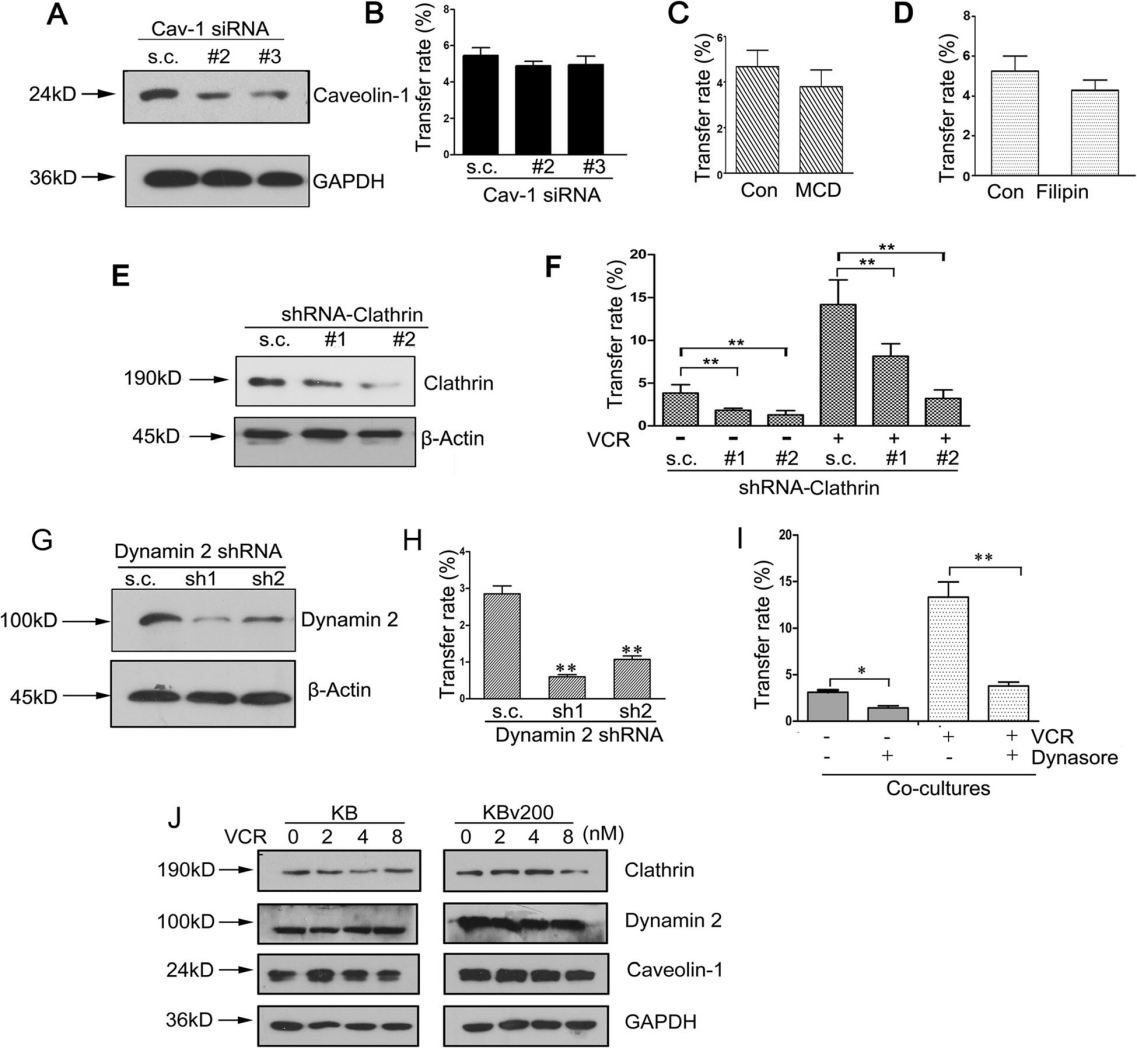
Dynamin 2 and Clathrin mediate the endocytosis of ABCB1-containing EV in recipient cancer cells **A** KB cells are transfected with siRNAs targeted against caveolin-1 and control scramble sequence RNA (s.c.). **B** The decrease of caveolin-1 expression in KB cells has no significant effect on the intercellular transfer of ABCB1 in co-cultures. **C-D** Pretreatments of KB cells with caveolae pharmacological inhibitors, methyl-β-cyclodextrin (MβCD; 1 mM)(C) and filipin III (D), show no remarkable inhibitory effects on ABCB1 transfer when compared with the untreated control cells. **E** The expression of clathrin is decreased by shRNA targeting clathrin as compared with control shRNA in KB cells. **F **The intercellular transfer rate of ABCB1 is decreased when the expression of clathrin is suppressed in KB cells. **G** Knockdown of dynamin2 using specific shRNA constructs is showed by immunoblot. **H** The suppression of dynamin-2 expression level in KB cells leads to a striking decrease in the intercellular transfer rate of ABCB1. **I** Pretreatment of KB cells with the inhibitor of dynamin GTPase Dynasore (10 μM) significantly reduces the rate of ABCB1 transfer, in the absence or presence of VCR. **J** Treatment with VCR did not alter the expression of clathrin, caveolin-1 and dynamin-2 in KB and KBv200 cells

The mechanism of EVs internalization was further investigated. Previous study showed two major endocytic pathways, a pathway associated with caveolin and a clathrin-mediated route, enable receptor internalization [22–24]. We pretreated recipient KB cells by transfecting siRNA against caveolin-1 following by co-culture with ABCB1-overexpressing KBv200 cells. But the obvious decrease of caveolin-1 expression had no significant effect on the intercellular transfer of ABCB1 in co-cultures, indicating caveolin-1 did not appear to be involved in the intercellular transfer of ABCB1 (Fig. 6, a-b). This was further evidenced by the fact that pretreatment of KB cells with two caveolin pharmacological inhibitors, filipin III (2.5 mg/L) and methyl-β-cyclodextrin (MβCD; 1 mM), showed no remarkable inhibitory effects on ABCB1 transfer when compared with the untreated control cells (Fig. 6, c-d). Whereas, when we suppressed the expression of clathrin in KB cells using two shRNA vectors, the intercellular transfer ratio of ABCB1 was decreased, either in the presence or absence of chemotherapeutic drug VCR, suggesting the involvement of clathrin-mediated endocytosis in the intercellular transfer of ABCB1 (Fig. 6, e-f).

Dynamin, is an important component of clathrin-mediated endocytosis, caveolar endocytosis, and other noncanonical endocytic processes [25]. To investigate further whether dynamin was indispensable for ABCB1 internalization, we decreased dynamin-2 expression in KB cells using two shRNA targeting dynamin-2 (Fig. 6, g). Flow cytometry analysis showed the suppression of intracellular dynamin-2 level in recipient cells led to a striking decrease in the intercellular transfer rate of ABCB1 (Fig. 6, h). We pretreated KB cells with a small-molecule inhibitor dynasore (10 μM) to block dynamin GTPase activities following by the cocultivation with KBv200 cells. It was showed that antagonism of dynamin GTPase activity by pretreatment of target cells with dynasore yielded a significant reduction of the rate of ABCB1 transfer, whether in the absence or presence of VCR (Fig. 6, i). This further demonstrated that dynamin 2-dependent endocytosis was involved in the intercellular transfer of ABCB1. However, treatment with VCR did not result in a significant alteration in the expression of caveolin-1, clathrin and dynamin-2, neither in donor cells nor in recipient cells (Fig. 6, j).

Collectively, these data suggest that, sensitive cancer cells could internalize these EVs by a clatherin- and dynamin 2-mediated manner, and accelerate ABCB1 recycling back to plasma membrane via decreasing the expression of Rab5.

### Chemotherapy-promoting intercellular transfer of ABCB1 occurs in vivo

**Fig. 7:**
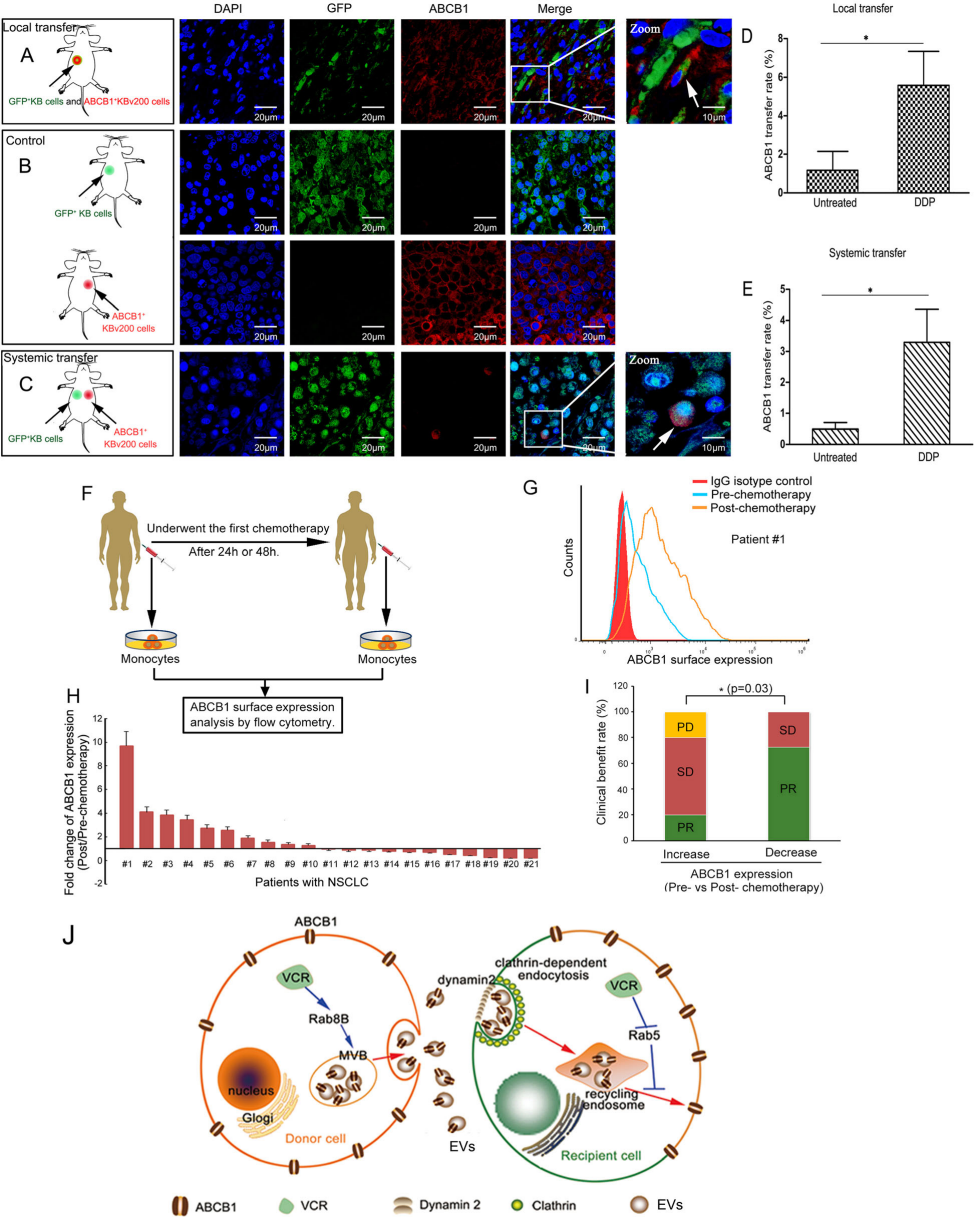
Chemotherapy-promoting intercellular transfer of ABCB1 occurs in vivo and is associated with chemotherapeutic efficacy in cancer patients. **A** The nude mice models are established by subcutaneously injecting a mixture of GFP^+^ KB cells and ABCB1^+^ KBv200 cells at a ratio of 1:1. Confocol images show the intercellular transfer of ABCB1 in xenograft tumors. Blue represents DAPI and red represents Cy3-ABCB1. **B** The nude mice models are established as control by subcutaneously injecting pure GFP+ KB cells or ABCB1+ KBv200 cells. **C** The systemic transfer models are generated by subcutaneously injecting GFP^+^ KB cells and KBv200 cells into the contralateral armpit of forelimb, respectively. Confocol images show the intercellular transfer of ABCB1 in xenograft tumors. **D-E** The mean percentage of ABCB1^+^ KB cells in isolated KB xenograft tumors is found to be elevated in mice receiving the treatment with DDP, as compared to that in untreated mice, whether in local mice model (D) or in systemic mice model (E). **F** ABCB1 surface expression was determined in peripheral blood monocyte subpopulations from patients with NSCLC before and after they received the first chemotherapy. **G-H** Each bar means the fold change of ABCB1 expression for each patient before and after chemotherapy. The bars above the horizontal axis represent the increase of ABCB1 expression after patients underwent chemotherapy. Ten cases of patients were found a rapid increase in ABCB1 surface expression. **I** Occurrence of ABCB1 intercellular transfer in patients predicted a poor chemotherapeutic efficacy. **J** When the heterogeneous tumor suffers the chemotherapy, ABCB1-overexpressing donor cancer cells release more EVs containing ABCB1 via the upregulation of Rab8B, then recipient sensitive cells take up those ABCB1-incorporated EVs through a clathrin-mediated dynamin 2-dependent endocytic pathway, following by a Rab5-mediated recycling back to plasma membrane. Hence the sensitive cancer cells instantly acquire the “short-term” resistance for evading the cytotoxicity of chemotherapeutics

In order to investigate whether ABCB1 intercellular transfer can occur in vivo and respond to chemotherapeutic drug, we established nude mice models by subcutaneously injecting a mixture of ABCB1 ^negative^ KB cells and ABCB1 ^positive^ KBv200 cells at a ratio of 1:1 (Fig. 7, a). Within 2 weeks, these mice developed palpable tumors. Then we examined the presence of KB cells obtaining ABCB1 molecules. Immunofluorescence staining showed ABCB1 ^positive^ KB cells were only occasionally observed in the xenograft tumors derived from mixed cells, rather than from another mice model that was established by only injected subcutaneously pure KB cells (Fig. 7, b, upper panel). This observation is in accordance with the hypothesis that intercellular transfer of ABCB1 occurs locally in vivo.

Next we tested the possibility of systemic transfer of ABCB1 by subcutaneously injecting GFP ^positive^ KB cells and KBv200 cells into the contralateral armpit of forelimb to establish xenograft tumors, respectively. We observed that individual KB cells acquired ABCB1 protein expression even when injected in contralateral tumors (Fig. 7, c). Surprisingly, the mean percentage of ABCB1 ^positive^ KB cells in isolated KB xenograft tumors was found to be elevated in mice receiving the treatment with DDP, as compared to that in untreated mice, whether in local mice model or in systemic mice model (Fig. 7, d-e). Yet, the efficiency of systemic transfer of ABCB1 was significantly lower than local transfer, presumably due to the deletion of direct cell-contact-dependent transfer. Overall, these data indicated intercellular transfer of ABCB1 probably locally or systemically responded to chemotherapy in vivo.

**Rapid alteration of ABCB1 expression may be a potential predictor of chemotherapeutic efficacy in cancer patients.**

To determine the correlation between ABCB1 expression alteration and therapeutic response, the ABCB1 expression level in peripheral blood monocytes of patients with non-small cell lung cancer (NSCLC) between pre- and post-chemotherapy was analyzed (Fig. 7, f). After these patients received 2 cycles of platinum-based regimen, chemotherapeutic efficacy was assessed. It was observed a variable increase of ABCB1 surface expression in the peripheral blood monocyte subpopulations after they received the first chemotherapy 24 h or 48 h in ten cases of patients (Fig. 7, g-h). Importantly, among these 10 patients, only 20% of patients achieved PR. In another 11 cases of patients who did not show a prompt increase of ABCB1 expression, 72.7% of patients achieved PR (Fig. 7, i). These data indicated quick increase of ABCB1 expression between pre- and post-chemotherapy may be associated with therapeutic efficacy.

## Discussion

MDR is a key concern in cancer management. Classical MDR is frequently attributed to the elevated expression of members of the ATP-binding cassette (ABC) transporters which are involved in drug detoxification and protection of tissues from xenobiotics [26–28]. Recent research showed extracellular vesicles (EVs) including exosomes, microvesicles and microparticles have been associated with the spread of drug-resistance. And EVs secreted by one drug-resistant cancer type can confer resistance on another cancer type [29]. But the effects of chemotherapeutic agents on the transmission of drug resistance mediated by EVs remain unclear. In this study, we found chemotherapeutic drugs significantly increased the amount of EVs released from drug-resistant cells. These EVs are enriched in ABCB1, which is a well-known MDR mediator. The recipient cells internalized these EVs and acquire the ability to efflux ABCB1 substrate Rho123, suggesting the successful delivery of functional ABCB1 protein. Thus, it is undeniable that EVs can serve as important mediators of the intercellular transfer of ABCB1. Generally, two key processes contribute to the EV-based intercellular protein transfer: the release of EVs by donor cells and the internalization of EVs by recipient cells. It’s worth noting that our data suggest more EVs will help more sensitive cells to gain the ability of efflux Rho123. And chemotherapeutic agents accelerated the recycling back to membrane of EVs in recipient cells by downregulating the Rab5. Since chemotherapeutic drugs also increased the secretion of EVs by upregulating the expression of Rab8b, it is easily concluded that chemotherapy facilitates sensitive cancer cells to develop quicker resistance by enhancing the secretion of EVs from drug-resistant cells and prompting the recycling of EVs in drug-sensitive cells.

Rab proteins, belonging to the Ras superfamily, are a large group of monomeric small GTPases. They are ubiquitously expressed and have been implicated in intracellular vesicle traffic through the recruitment of effectors. Rab8, which has two isoforms, Rab8A and Rab8B, belongs to a subset of Rabs that contain a CAAX motif. This is most similar to Rab10 and Rab13. To date, researchers has suggested Rab8 promotes polarized membrane transport linked cell morphogenesis [30, 31]. A recent study showed that Rab8 play an essential role in West Nile Virus particle release and transport [32].Rab8B can act as a positive regulator of Wnt/ β-catenin and is required for LRP6 endocytosis and activity [33]. And Rab8B interact with TRIP8b and is involved in the regulated secretory pathway in AtT20 cells [34]. However, up to now, no evidence has found that Rab8B associate with tumor MDR. In fact, most of research focused on Rab8, they did not distinguish between Rab8A and Rab8B in the limited studies. Very little is known about the role and mechanism of Rab8B in tumor cells. For the first time, our study demonstrated that Rab8B is associated with the development of urgent MDR. Inhibition of Rab8B expression reduced the secretory amount of ABCB1-enriched EVs, consequently, ABCB1 intercellular transfer was significantly decreased. Given that, combination Rab8B inhibitors and conventional chemotherapeutic drugs may be potential way to circumvent MDR and improve the efficacy of chemotherapy.

Accumulating evidence showed that the plasma membrane localization of ABCB1 can be readily modified by modulating its endocytic/recycling traffic, which involves the regulation of Rab GTPases. Among many of the endosomal Rab proteins, Rab5 are widely known to regulate ABCB1 trafficking and recycling in cancer cells [35, 36]. Our results also showed the suppression of Rab5 resulted in an enhanced surface localization of ABCB1 in recipient cells. And, crucially, the treatment with VCR obviously decreased the expression of Rab5 in recipient cells. Presumably, the treatment with VCR appears to enhance the recycling back to plasma membrane of ABCB1, after EVs were endocytosed by recipient cancer cells. Thus, we conclude that the downregulation of small GTPase Rab5 in recipient cells upon the treatment of chemotherapeutics could be an important molecular event associated with the increased transfer efficiency of ABCB1. But the potential roles of other Rab proteins in the intercellular transfer of ABCB1 promoted by chemotherapeutic agents still needs further investigations.

Recently, several studies proposed a new way to spread drug resistance within a cancer cell population, that was intercellular transfer of ABCB1 [37]. In the present study, we evidenced the existence of intercellular ABCB1 transfer from drug-resistant cancer cells to sensitive cancer cells. Importantly, the recipient cells acquired an urgent resistant phenotype, which manifested as decreased susceptibility to chemotherapeutic drug Dox and reduced intracellular accumulating level of Rho123 or Dox. These data highly reinforce the fact that, ABCB1 can be transferred from a donor cell to a recipient one in vitro, keeping its ability to efflux drugs, and thus, conferring MDR phenotype to the recipient cells. In addition, this acquisition of MDR phenotype did not involve the exchange or induction of mRNA, because there was no significant alteration in the expression of ABCB1 mRNA in sensitive cancer cells. The intercellular transfer of ABCB1 was also observed between GFP-expressing drug-sensitive human colon carcinoma cells S1 and KBv200 cells. It indicated that ABCB1 transfer occurred in the cells of different origin. It has been reported that ABCB1 intercellular transfer occurred in leukemia cells [18], osteosarcoma [38], neuroblastoma and breast adenocarcinoma cells [16]. These studies indicate ABCB1 intercellular transfer may be not tumor-specific. Our results supported the theory that there was a nongenetic mechanism in the acquisition of MDR. In particular, we presented here that transient exposure of chemotherapeutic drugs, regardless of whether the agents are transported by ABCB1, significantly stimulated the functional transfer of ABCB1 from resistant donor cells to sensitive recipient cells without induced expression of *ABCB1* gene. More fascinatingly, the intercellular transfer of ABCB1 was observed to occur and respond to chemotherapeutics in vivo, which was demonstrated by establishing homolateral or contralateral xenograft tumors with sensitive KB and resistant KBv200 cells in nude mice. To the best of our knowledge, this is the first report showing the relevance of chemotherapeutic drugs and ABCB1 transfer, both in vitro and in vivo. This is conducive to understand why sensitive cancer cells can promptly acquire a survival advantage in the context of chemotherapeutics.

To date, several reports have identified ABCB1 gene overexpression as an independent negative prognostic factor in clinical outcome [39–41]. It has been observed that the proportion of tumor cells expressing ABCB1 increased in some metastatic tumors from patients that have undergone multiple cycles of chemotherapy. However, no clinical study is available to demonstrate the occurrence of ABCB1 transfer. In this study, some newly diagnosed patients with non-small cell lung cancer was observed a rapid increase of the membrane expression level of ABCB1 in peripheral blood monocytes, after they received the first course of chemotherapy. The prompt increase of ABCB1 expression seems to be associated with poor chemotherapeutic response. These data implied rapid alteration of ABCB1 expression may be a potential predictor of chemotherapeutic efficacy. But this needs more investigations in more cases of patient with NSCLC, or other types of cancer. Since the intercellular transfer of ABCB1 occurs in different types of tumors, and in cells of different origin, it would have important implications to clarify whether ABCB1 transfer occurs in tumor patients and the relationship with chemotherapeutic response.

## Conclusions

In summary, our findings suggest a new mechanism of how chemotherapeutic drugs assist sensitive cancer cells in acquiring an urgent resistance. Chemotherapeutic drugs elicit the secretion and recycling of EVs by dysregulating Rab8B and Rab5, leading to a significant increase of ABCB1 intercellular transfer (Fig. 7, j). This is the first to demonstrate that Rab8B contributes to the development of MDR. This implied that combining inhibition of Rab8B and Rab5 with conventional chemotherapeutic agents would be a potential way to improve chemotherapy effectiveness.

## Supplementary information

**Additional file 1: Figure. S1.** **a** The mRNA expression of ABCB1 was not detectable in KB cells with incubation of EVs by RT-PCR. **b-c** Treatment with DDP results in an obvious concentration-dependent increase in the percentage of KB cells with negative rhodamine 123 staining when the KB cells are cocultured with equal EVs.

**Additional file 2: Figure. S2. a-b** The effect of Dox on the intercellular transfer of ABCB1 in co-cultures is detemined by flow cytometry. **c-d** Representative flow cytometric analysis shows VCR and DDP could not increase the surface expression of ABCB1 in sensitive KB cells in short-term culture. **e** Intercellular transfer of ABCB1 was found between S1 cells and KBv200 cells. 

**Additional file 3: Figure. S3.** The IC_50_ values of Dox **a** and DDP **b** in the indicated cells are showed. **c** The IC_50_ values of Dox in the absence or presence of verapamil (VRP) in the indicated cells are showed. **d** The IC_50_ values of Dox in Aq-MDR cells are long-termly examined by MTT assays.

**Additional file 4: Table S1.** Primer sequences for PCR. **Table S2.** The efficient targeting seqences for specific genes are shown.

## Data Availability

The key raw data are available on the Research Data Deposit public platform (www.researchdata.org.cn, RDDB20190006).

## Abbreviations

ABCB1: ATP Binding Cassette Subfamily B Member 1
DDP: Cisplatin
Dox: Doxorubicin
EVs: Extracellular vesicles
MDR: Multi-drug resistance
MβCD: Methyl-β-cyclodextrin
NSCLC: Non-small-cell lung carcinoma
VCR: Vincristine
