# Supplementary figures and images for: Chemotherapeutic drugs stimulate the release and recycling of extracellular vesicles to assist cancer cells in developing an urgent chemoresistance
Source: Mol Cancer. 2019 Dec 12;18:182. doi: 10.1186/s12943-019-1114-z (PMC6907227; doi:10.1186/s12943-019-1114-z)

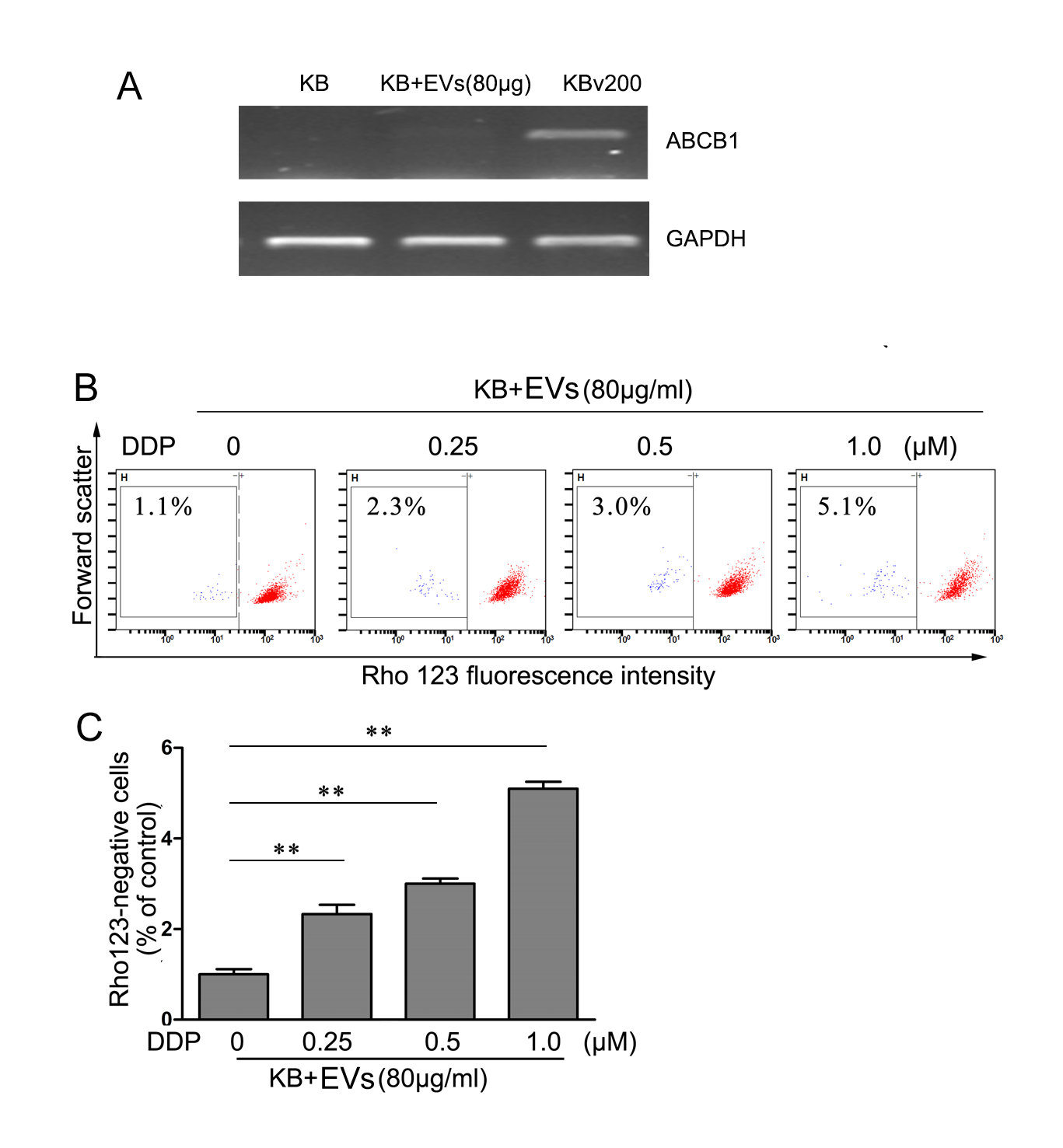

Supplement: Supplementary file 1 — Additional file 1: Figure. S1. a The mRNA expression of ABCB1 was not detectable in KB cells with incubation of EVs by RT-PCR. b-c Treatment with DDP results in an obvious concentration-dependent increase in the percentage of KB cells with negative rhodamine 123 staining when the KB cells are cocultured with equal EVs. [file 12943_2019_1114_MOESM1_ESM.tif]

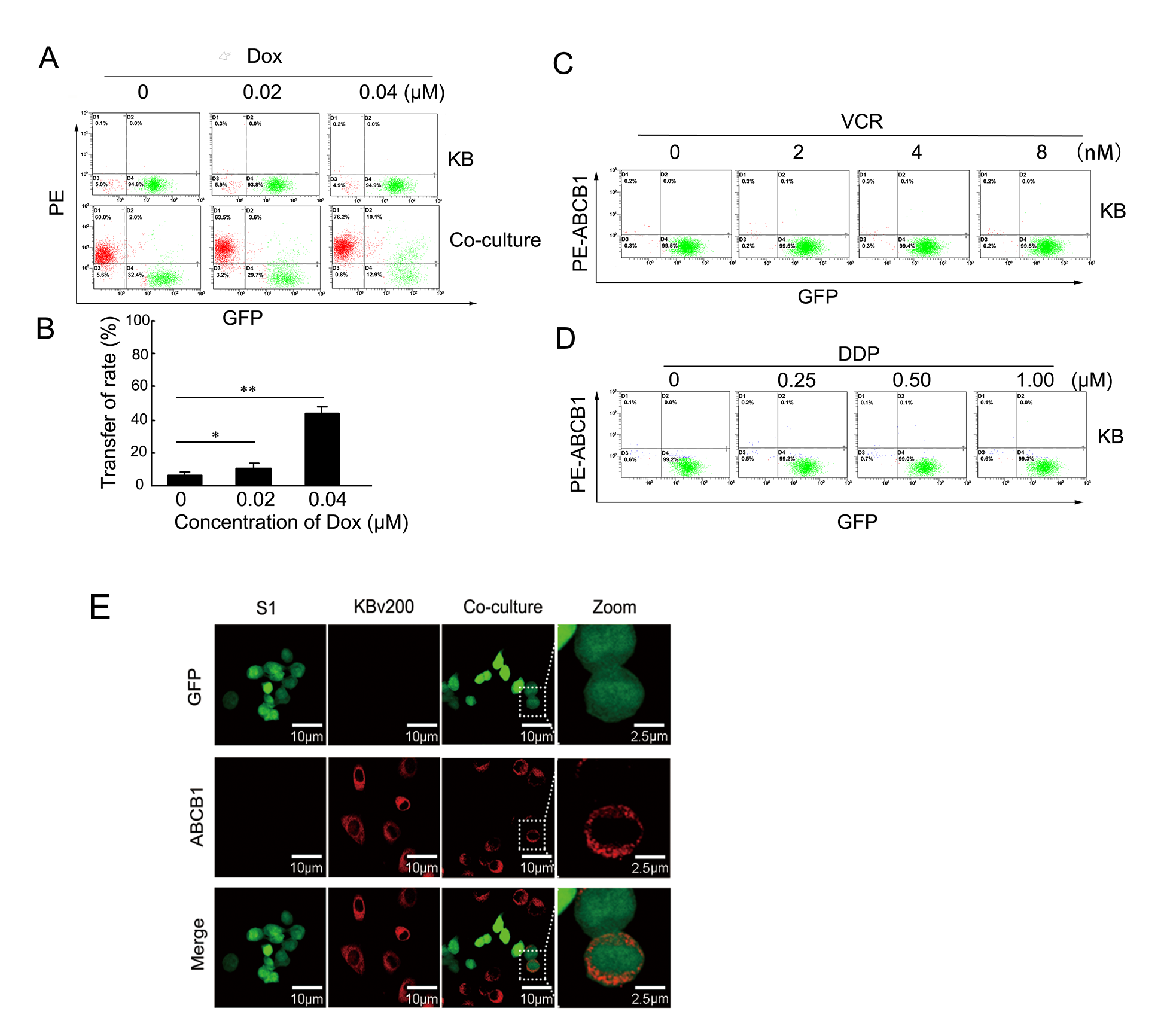

Supplement: Supplementary file 2 — Additional file 2: Figure. S2. a-b The effect of Dox on the intercellular transfer of ABCB1 in co-cultures is detemined by flow cytometry. c-d Representative flow cytometric analysis shows VCR and DDP could not increase the surface expression of ABCB1 in sensitive KB cells in short-term culture. e Intercellular transfer of ABCB1 was found between S1 cells and KBv200 cells. [file 12943_2019_1114_MOESM2_ESM.tif]

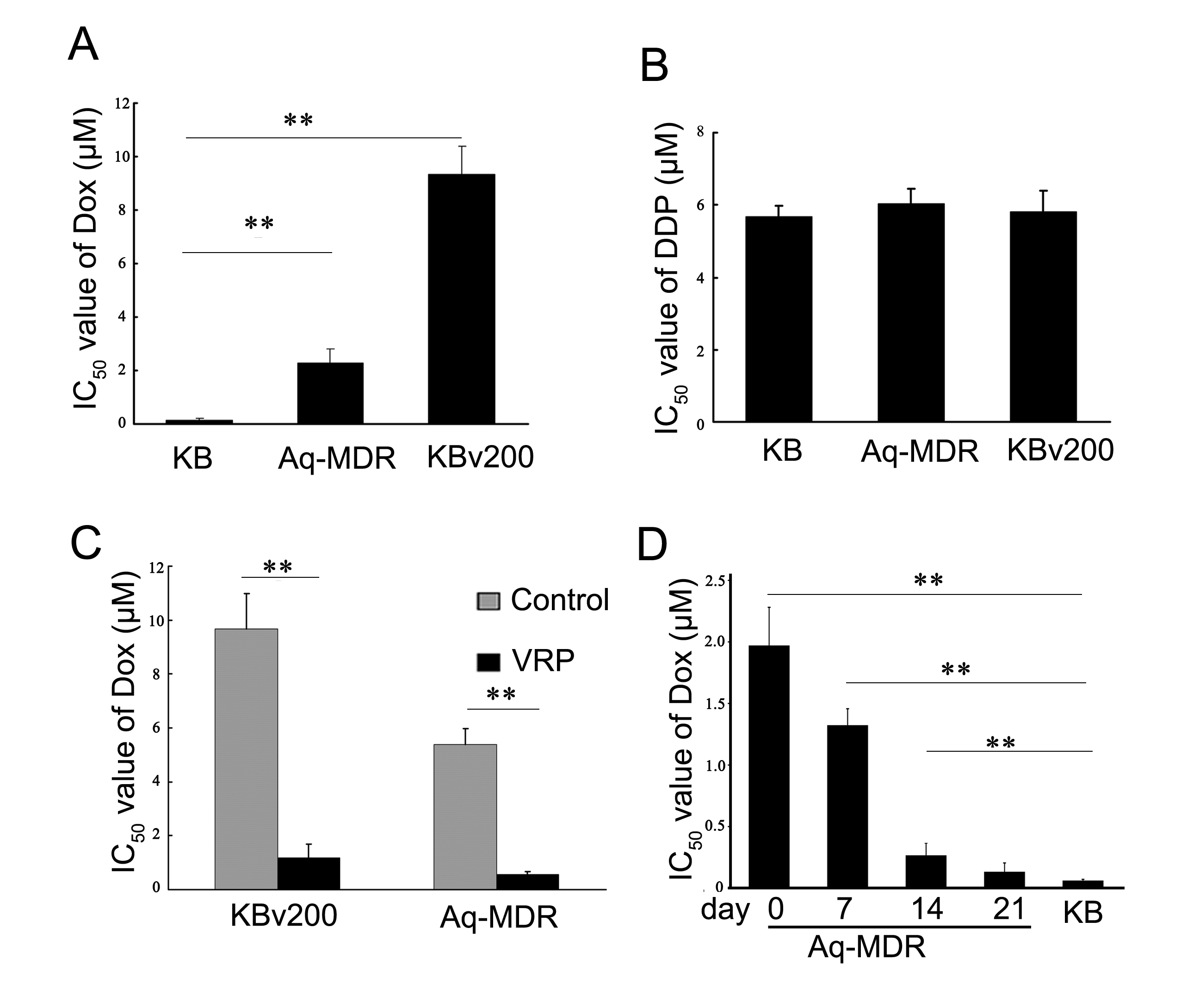

Supplement: Supplementary file 3 — Additional file 3: Figure. S3. The IC50 values of Dox a and DDP b in the indicated cells are showed. c The IC50 values of Dox in the absence or presence of verapamil (VRP) in the indicated cells are showed. d The IC50 values of Dox in Aq-MDR cells are long-termly examined by MTT assays. [file 12943_2019_1114_MOESM3_ESM.tif]
