# Supplementary material for: Chemotherapeutic drugs stimulate the release and recycling of extracellular vesicles to assist cancer cells in developing an urgent chemoresistance
Source: Mol Cancer. 2019 Dec 12;18:182. doi: 10.1186/s12943-019-1114-z (PMC6907227; doi:10.1186/s12943-019-1114-z)
Supplement: Supplementary file 4 — Additional file 4: Table S1. Primer sequences for PCR. Table S2. The efficient targeting seqences for specific genes are shown. [file 12943_2019_1114_MOESM4_ESM.doc]

**Supplementary figure legends**

**Figure S1.** (A-B)Treatment with DDP results in an obvious concentration-dependent increase in the percentage of KB cells with negative rhodamine 123 staining when the KB cells are cocultured with equal EVs.

**Figure S2.** (A-B) The effect of Dox on the intercellular transfer of ABCB1 in co-cultures is detemined by flow cytometry. (C-D) Representative flow cytometric analysis shows VCR and DDP could not increase the surface expression of ABCB1 in sensitive KB cells in short-term culture.

**Figure S3.** The IC_50_ values of Dox **(A**) and DDP (B) in the indicated cells are showed. (C) The IC_50_ values of Dox in the absence or presence of verapamil (VRP) in the indicated cells are showed. (D) The IC_50_ values of Dox in Aq-MDR cells are long-termly examined by MTT assays.

**Table S1. Primer sequences for PCR.**

| Primer | Sequence |
| --- | --- |
| ABCB1-F | 5′- GTGGG-GCAAGTCAGTTCATT -3′ |
| ABCB1-R | 5′- TCTTCACCTCCAGGCTCAGT -3 |
| GAPDH-F | 5′-GAGTCAACGGATTTGGTCGT - 3′ |
| GAPDH-R | 5′- GATCTCGCTCCTGGAAGATG -3′ |
| Rab1-F | 5′- ATGTCCAGCATGAATCCCGAA-3′ |
| Rab1-R | 5′-AAGAAGGCAAGACTTTCCAACCC -3′ |
| Rab3D-F | 5′- GGGAAATTGCCATGACCACA-3′ |
| Rab3D-R | 5′-TCCCGTTAACACTGAGACCAC -3′ |
| Rab4A-F | 5′- CTTAGAAGCCTCCAGATTTGC-3′ |
| Rab4A-R | 5′-GCATCTCCGTACTGAATACCTG -3′ |
| Rab4B-F | 5′- GTCATGGCTGAGACCTACGACT-3′ |
| Rab4B-R | 5′-GATCCAAACTCCACGCCGAT -3′ |
| Rab5A-F | 5′-AACGATACCATAGCCTAGCAC -3′ |
| Rab5A-R | 5′- CTTGCTTGCCTCTGAAGTTCT-3′ |
| Rab7-F | 5′-CAAAACTCCCGTTAGATCAGC -3′ |
| Rab7-R | 5′-CTCCGCTAACCTAAGAATACCACA -3′ |
| Rab8A-F | 5′-CGCTATCTCCAAATCGGACGTT-3′ |
| Rab8A-R | 5′- GACAGAATCGCTTCATGGCACT-3′ |
| Rab8B-F | 5′-AGCCTCTAGGAAAAGTCTTGC -3′ |
| Rab8B-R | 5′-CTTTAGCCCAAATGACTGGAC -3′ |
| Rab10-F | 5′-TTCAAGCTGCTCCTGATCGG -3′ |
| Rab10-R | 5′-CTGGCCTGCTGTATCCCATAT -3′ |
| Rab11B-F | 5′-CCTTCATCGAGACCTCAGCCTT -3′ |
| Rab11B-R | 5′-CACGCTGATGTCCACCACGTT -3′ |
| Rab12-F | 5′-TCTGCCACGAAGCAAAGCTC -3′ |
| Rab12-R | 5′-GGTACATCCCTTAGGAAGCCAT -3′ |
| Rab13-F | 5′- GCCTACGACCACCTCTTCAAG-3′ |
| Rab13-R | 5′-TTCCCCTCTATATCCACAGTGC -3′ |
| Rab20-F | 5′- ATCCAGTCATAAGCCACCCAA-3′ |
| Rab20-R | 5′-ATAGCCAGCCATCATTTCCACT -3′ |
| Rab22-F | 5′-CTTGAGTCTTCTCCGTGCAAA -3′ |
| Rab22-R | 5′-AAGCCAACGATGTAGTCCCTT -3′ |
| Rab25-F | 5′- GCACCTACCTAATGAACAGGAC-3′ |
| Rab25-R | 5′- ATCATCCAGTTAGCCAGGAGT-3′ |
| Rab26-F | 5′-AGGCTCTGGCTCAAACAGCAA -3′ |
| Rab26-R | 5′-TCGGAAATCACAGACCTCACCT -3′ |
| Rab27A-F | 5′-CCGCTATATTCCCATTGCTCT -3′ |
| Rab27A-R | 5′- ATAACTCGGCATAAGCACCAG-3′ |
| Rab27B-F | 5′- GCTCCTTGTGAAAACACCTA-3′ |
| Rab27B-R | 5′- ACATATCTCCTTTGAGCCTGAC-3′ |
| Rab35-F | 5′-CGAGTCCTTTGTCAACGTCAAG -3′ |
| Rab35-R | 5′-TTTGTAGGCATCTTCCGTCT -3′ |

| Accession number | Gene name | Duplex # | Sequence |
| --- | --- | --- | --- |
| NM_001833 | Clathrin | 1 | GGAAAGTAATGGTCCAACA |
|  |  | 2 | GAAAGTAATGGTCCAACAG |
|  |  | 3 | CCAATTCTCGGAAGCAAGA |
| NM_004945 | Dynamin-2 | 1 | CCGAATCAATCGCATCTTC |
|  |  | 2 | GACATGATCCTGCAGTTCA |
|  |  | 3 | CCTCCGAGCTGGCGTCTAC |
| NM_004162 | Rab5 | 1 | GGAAGAGGAGTAGACCTTA |
|  |  | 2 | AGGAATCAGTGTTGTAGTA |
|  |  | 3 | GAAGAGGAGTAGACCTTAC |
| NM_ 016530 | Rab8B | 3 | CCTGGGTAACAAATGTGATAT |
|  |  | 4 | CGAAAGAATGATCCTGGGTAA |
|  |  | 5 | GCTAGCAATTGACTATGGGAT |
| NM_001003296 | Caveolin-1 | 1 | CCTTCACTGTGACGAAATA |
|  |  | 2 | GCAGTTGTACCATGCATTA |
|  |  | 3 | GCGACCCTAAACACCTCAA |

**Table S2. The efficient targeting seqences for specific genes are shown.**
